# Supplementary material for: Novel complete methanogenic pathways in longitudinal genomic study of monogastric age-associated archaea
Source: Anim Microbiome. 2023 Jul 17;5:35. doi: 10.1186/s42523-023-00256-6 (PMC10353118; doi:10.1186/s42523-023-00256-6)
Supplement: Supplementary file 3 — Additional file 3: Fig. S1. Complete, non-collapsed, Methanobacteriales and Methanomassiliicoccales phylogenetic trees with statistics. [file 42523_2023_256_MOESM3_ESM.pdf]

Phylogenetic Tree Report

Rendered Tree

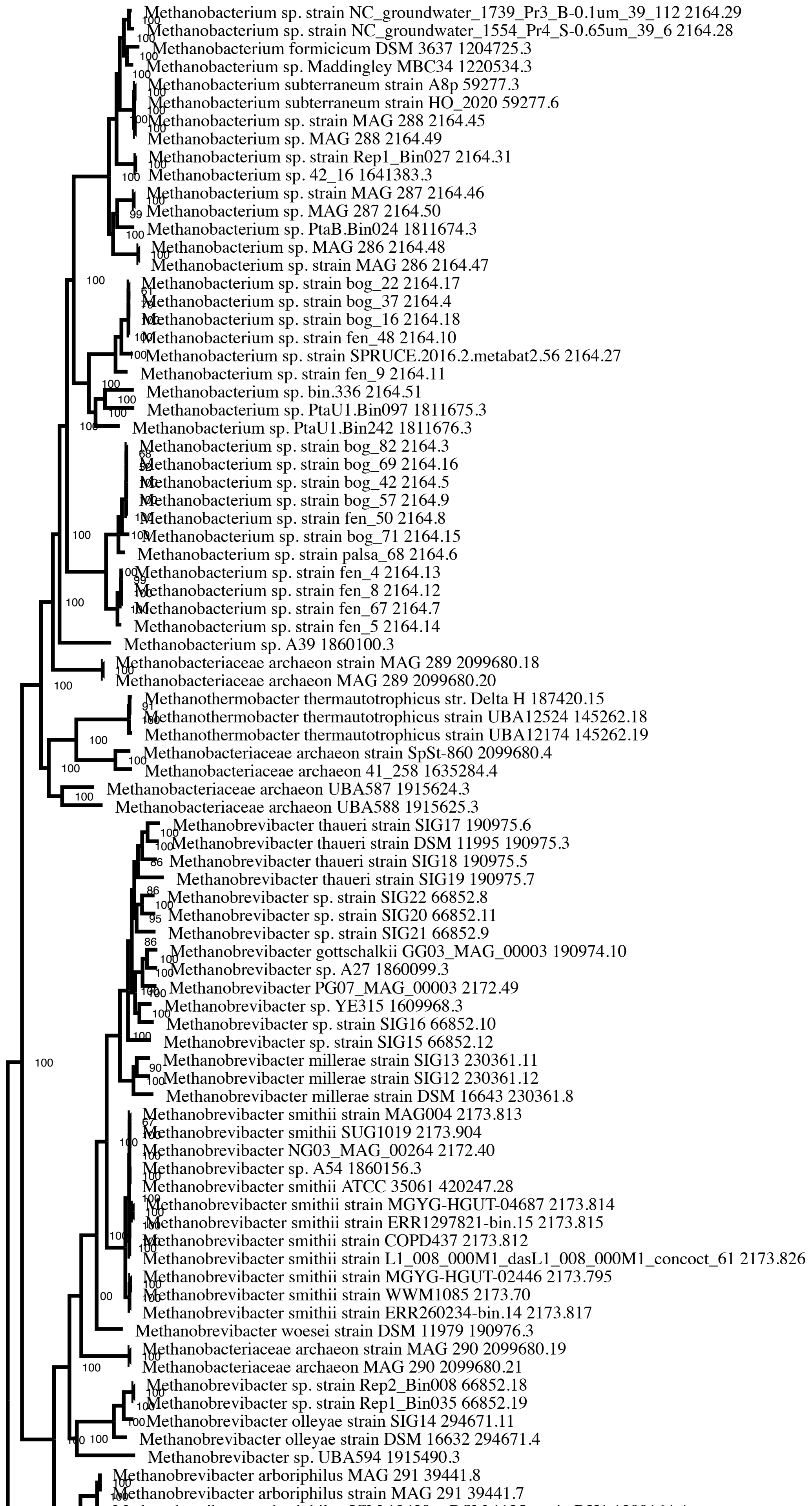

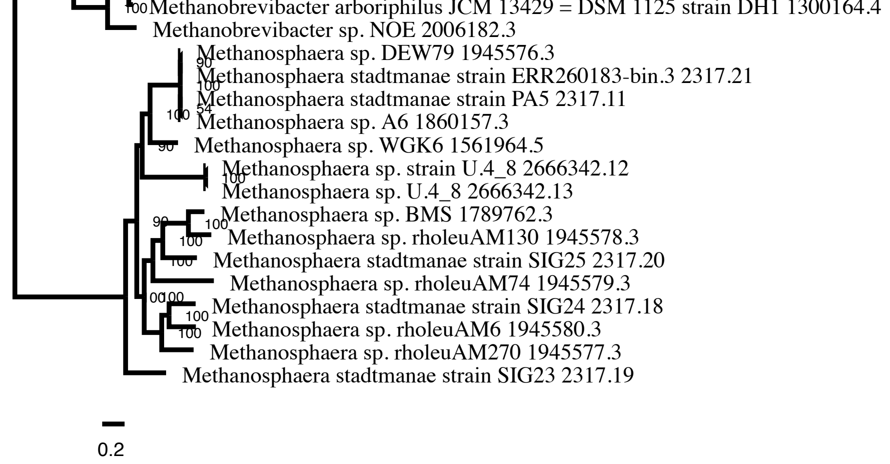

Alternate View

Files with more details

Tree Analysis Statistics

**Requested genomes**	100
**Genomes with data**	100
**Max allowed deletions**	10
**Max allowed duplications**	10
**Single-copy genes requested**	100
**Single-copy genes found**	100
**Num protein alignments**	100
**Alignment program**	mafft
**Protein alignment time**	2245.4 seconds
**Num aligned amino acids**	38462
**Num CDS alignments**	96
**Num aligned nucleotides**	111951
**Best protein model found by RAxML**	LG
**Branch support method**	RAxML Fast Bootstrapping
**RAxML likelihood**	-4785827.9582
**RAxML version**	8.2.11
**RAxML time**	116639.0 seconds
**Total time**	119167.4 seconds

RAxML Command Line

Goal: Analyze proteins with model 'AUTO' to find best substitution model.
raxmlHPC-PTHREADS-SSE3 -s Methanobacteriaceae_10_MAX_DELs_DUPs_proteins.phy -n Methanobacteriaceae_10_MAX_DELs_DUPs_proteins -m PROTCATAUTO -p 12345 -T 12 -e 10
Process time: 20294.419 seconds

Goal: Find best tree.
raxmlHPC-PTHREADS-SSE3 -s Methanobacteriaceae_10_MAX_DELs_DUPs.phy -n Methanobacteriaceae_10_MAX_DELs_DUPs -m GTRCAT -q Methanobacteriaceae_10_MAX_DELs_DUPs.partitions -p 12345 -T 12 -f a -x 12345 -N 100
Process time: 96344.551 seconds

RAxML Codon and Amino Acid Partitions

```
DNA, codon1 = 1-111951\3
DNA, codon2 = 2-111951\3
DNA, codon3 = 3-111951\3
LG, proteins = 111952-150413
```

RAxML Warnings

Sequences 2099680.18 and 2099680.20 are exactly identical
Sequences 2164.45 and 2164.49 are exactly identical
Sequences 2164.46 and 2164.50 are exactly identical
Sequences 2164.47 and 2164.48 are exactly identical
Sequences 39441.7 and 39441.8 are exactly identical

Genome Statistics

GenomeId	Total Genes	Single Copy	Used	Name
190974.10	13	5	1	Methanobrevibacter gottschalkii GG03_MAG_00003
1945577.3	738	248	80	Methanosphaera sp. rholeuAM270
1945579.3	893	284	94	Methanosphaera sp. rholeuAM74
2666342.12	901	298	96	Methanosphaera sp. strain U.4_8
2666342.13	901	298	96	Methanosphaera sp. U.4_8
2172.40	932	228	72	Methanobrevibacter NG03_MAG_00264
2006182.3	953	305	97	Methanobrevibacter sp. NOE
1915625.3	953	269	86	Methanobacteriaceae archaeon UBA588
1945578.3	961	305	98	Methanosphaera sp. rholeuAM130
2099680.4	984	307	99	Methanobacteriaceae archaeon strain SpSt-860
2099680.19	985	316	100	Methanobacteriaceae archaeon strain MAG 290
2099680.21	985	316	100	Methanobacteriaceae archaeon MAG 290
66852.12	995	281	93	Methanobrevibacter sp. strain SIG15
2099680.18	997	284	94	Methanobacteriaceae archaeon strain MAG 289
2099680.20	997	284	94	Methanobacteriaceae archaeon MAG 289
1811676.3	1013	251	84	Methanobacterium sp. PtaU1.Bin242
2317.19	1035	299	98	Methanosphaera stadtmanae strain SIG23
1635284.4	1052	309	99	Methanobacteriaceae archaeon 41_258
1945580.3	1071	314	98	Methanosphaera sp. rholeuAM6
1789762.3	1076	317	100	Methanosphaera sp. BMS
2164.14	1078	268	84	Methanobacterium sp. strain fen_5
2164.11	1084	255	87	Methanobacterium sp. strain fen_9
2317.18	1100	314	99	Methanosphaera stadtmanae strain SIG24
2164.7	1103	271	84	Methanobacterium sp. strain fen_67
2317.20	1107	318	100	Methanosphaera stadtmanae strain SIG25
1915624.3	1112	284	93	Methanobacteriaceae archaeon UBA587
1561964.5	1144	318	99	Methanosphaera sp. WGK6
66852.19	1152	282	93	Methanobrevibacter sp. strain Rep1_Bin035
1915490.3	1158	224	72	Methanobrevibacter sp. UBA594
2317.21	1161	295	93	Methanosphaera stadtmanae strain ERR260183-bin.3
145262.18	1171	258	81	Methanothermobacter thermautotrophicus strain UBA12524
2164.51	1199	307	95	Methanobacterium sp. bin.336
190975.7	1214	293	94	Methanobrevibacter thaueri strain SIG19
2164.48	1217	304	94	Methanobacterium sp. MAG 286
2164.47	1217	304	94	Methanobacterium sp. strain MAG 286
2172.49	1285	318	100	Methanobrevibacter PG07_MAG_00003
66852.10	1296	309	97	Methanobrevibacter sp. strain SIG16
230361.11	1296	285	91	Methanobrevibacter millerae strain SIG13
66852.8	1316	288	89	Methanobrevibacter sp. strain SIG22
2164.50	1317	300	97	Methanobacterium sp. MAG 287
2164.46	1317	300	97	Methanobacterium sp. strain MAG 287
1945576.3	1318	318	100	Methanosphaera sp. DEW79
2164.12	1322	320	100	Methanobacterium sp. strain fen_8
2164.13	1324	315	99	Methanobacterium sp. strain fen_4
1860157.3	1325	306	98	Methanosphaera sp. A6
190975.6	1352	306	97	Methanobrevibacter thaueri strain SIG17
2317.11	1357	319	100	Methanosphaera stadtmanae strain PA5

2164.28	1378	260	86	Methanobacterium sp. strain NC_groundwater_1554_Pr4_S-0.65um_39_6
294671.11	1390	311	100	Methanobrevibacter olleyae strain SIG14
145262.19	1396	284	92	Methanothermobacter thermautotrophicus strain UBA12174
1811674.3	1407	320	100	Methanobacterium sp. PtaB.Bin024
66852.18	1411	312	100	Methanobrevibacter sp. strain Rep2_Bin008
190975.5	1415	305	97	Methanobrevibacter thaueri strain SIG18
230361.8	1422	320	100	Methanobrevibacter millerae strain DSM 16643
2173.904	1437	318	100	Methanobrevibacter smithii SUG1019
66852.9	1437	294	92	Methanobrevibacter sp. strain SIG21
2164.27	1438	315	98	Methanobacterium sp. strain SPRUCE.2016.2.metabat2.56
230361.12	1439	318	100	Methanobrevibacter millerae strain SIG12
66852.11	1445	320	100	Methanobrevibacter sp. strain SIG20
2173.813	1445	320	100	Methanobrevibacter smithii strain MAG004
39441.8	1446	304	98	Methanobrevibacter arboriphilus MAG 291
39441.7	1446	304	98	Methanobrevibacter arboriphilus strain MAG 291
190976.3	1456	320	100	Methanobrevibacter woesei strain DSM 11979
2164.15	1475	294	92	Methanobacterium sp. strain bog_71
2173.826	1514	320	100	Methanobrevibacter smithii strain L1_008_000M1_dasL1_008_000M1_concoct_61
1860156.3	1529	312	99	Methanobrevibacter sp. A54
2173.812	1536	317	100	Methanobrevibacter smithii strain COPD437
294671.4	1540	314	100	Methanobrevibacter olleyae strain DSM 16632
2173.817	1550	320	100	Methanobrevibacter smithii strain ERR260234-bin.14
420247.28	1575	320	100	Methanobrevibacter smithii ATCC 35061
2173.795	1602	320	100	Methanobrevibacter smithii strain MGYG-HGUT-02446
2173.70	1619	320	100	Methanobrevibacter smithii strain WWM1085
1860099.3	1648	314	98	Methanobrevibacter sp. A27
2164.6	1653	313	100	Methanobacterium sp. strain palsa_68
187420.15	1665	319	100	Methanothermobacter thermautotrophicus str. Delta H
1811675.3	1724	306	96	Methanobacterium sp. PtaU1.Bin097
1300164.4	1753	319	100	Methanobrevibacter arboriphilus JCM 13429 = DSM 1125 strain DH1
2164.10	1759	300	95	Methanobacterium sp. strain fen_48
2164.29	1784	319	100	Methanobacterium sp. strain NC_groundwater_1739_Pr3_B-0.1um_39_112
2164.18	1785	307	98	Methanobacterium sp. strain bog_16
2164.8	1792	304	98	Methanobacterium sp. strain fen_50
1609968.3	1798	320	100	Methanobrevibacter sp. YE315
190975.3	1857	320	100	Methanobrevibacter thaueri strain DSM 11995
2164.31	1894	320	100	Methanobacterium sp. strain Rep1_Bin027
2164.17	1910	319	100	Methanobacterium sp. strain bog_22
2164.16	1950	316	99	Methanobacterium sp. strain bog_69
2164.49	1989	319	100	Methanobacterium sp. MAG 288
2164.45	1989	319	100	Methanobacterium sp. strain MAG 288
59277.6	2003	319	100	Methanobacterium subterraneum strain HO_2020
2173.814	2013	223	68	Methanobrevibacter smithii strain MGYG-HGUT-04687
2173.815	2013	223	68	Methanobrevibacter smithii strain ERR1297821-bin.15
2164.9	2018	314	99	Methanobacterium sp. strain bog_57
1641383.3	2046	312	98	Methanobacterium sp. 42_16
1220534.3	2072	309	95	Methanobacterium sp. Maddingley MBC34
2164.4	2114	308	97	Methanobacterium sp. strain bog_37
2164.5	2136	312	99	Methanobacterium sp. strain bog_42
2164.3	2198	317	99	Methanobacterium sp. strain bog_82
1204725.3	2203	320	100	Methanobacterium formicicum DSM 3637
59277.3	2227	319	100	Methanobacterium subterraneum strain A8p
1860100.3	2857	319	100	Methanobacterium sp. A39

Gene Family Statistics

Gene families are ranked by alignment score combining mean per-position variability, alignment length, and gappiness.

PGFam	Align. Score	Align. Length	Num Seqs	Mean Sqr Freq	Prop Gaps	Used In Analysis	Product
PGF_01175575	15.09	630	99	0.601	0.069	True	Threonyl-tRNA synthetase (EC 6.1.1.3)
PGF_05070366	14.92	573	96	0.623	0.077	True	Dihydroxy-acid dehydratase (EC 4.2.1.9)
PGF_00018085	14.73	555	95	0.625	0.084	True	Lysyl-tRNA synthetase (class I) (EC 6.1.1.6)
PGF_00065138	14.39	420	97	0.702	0.076	True	Uncharacterized protein MJ0800
PGF_00421761	14.24	321	96	0.795	0.054	True	DNA repair and recombination protein RadA
PGF_00052238	14.05	454	99	0.659	0.070	True	Signal recognition particle protein Ffh
PGF_03609651	14.00	720	97	0.522	0.148	True	Methionyl-tRNA synthetase (EC 6.1.1.10)
PGF_00424924	13.73	418	94	0.672	0.047	True	Eukaryotic peptide chain release factor subunit 1
PGF_00012928	13.36	378	92	0.687	0.106	True	Hydroxymethylglutaryl-CoA synthase (EC 2.3.3.10)
PGF_00426105	13.26	439	97	0.633	0.129	True	5,6,7,8-tetrahydromethanopterin hydro-lyase (EC 4.2.1.147) @ Formaldehyde activating enzyme / D-arabino-3-hexulose 6-phosphate formaldehyde-lyase (EC 4.1.2.43)
PGF_00012505	13.25	492	97	0.597	0.097	True	Hydrogenase expression/formation protein
PGF_10373476	13.25	442	97	0.630	0.067	True	Diaminopimelate decarboxylase (EC 4.1.1.20)
PGF_09019918	13.24	436	92	0.634	0.043	True	Glutamate-1-semialdehyde 2,1-aminomutase (EC 5.4.3.8)
PGF_03316046	13.23	464	96	0.614	0.176	True	Threonine synthase (EC 4.2.3.1)
PGF_00012926	13.23	413	93	0.651	0.042	True	Hydroxymethylglutaryl-CoA reductase (EC 1.1.1.34)
PGF_00015813	13.22	722	98	0.492	0.176	True	Uncharacterized KH and PIN-domain containing protein MJ1533
PGF_03590193	13.18	445	98	0.625	0.087	True	Serine hydroxymethyltransferase (tetrahydromethanopterin-dependent)
PGF_02620298	13.10	484	93	0.595	0.058	True	Argininosuccinate lyase (EC 4.3.2.1)
PGF_07750515	13.08	455	95	0.613	0.067	True	Phosphoribosylamine--glycine ligase (EC 6.3.4.13)
PGF_00065212	12.74	429	96	0.615	0.076	True	Uncharacterized protein MJ1313
PGF_00006244	12.73	387	98	0.647	0.107	True	Formate--phosphoribosylaminoimidazolecarboxamide ligase (EC 6.3.4.23)
PGF_00064425	12.57	453	95	0.591	0.098	True	UbiD family decarboxylase, MJ1133 type
PGF_00008338	12.51	463	95	0.581	0.082	True	Glutamyl-tRNA(Gln) amidotransferase asparaginase subunit (EC 6.3.5.7)
PGF_03060918	12.40	406	93	0.615	0.064	True	Uncharacterized protein SSO2743
PGF_04375438	12.28	420	94	0.599	0.115	True	Coenzyme B synthesis from 2-oxoglutarate: steps 1, 6, and 10
PGF_00421409	12.18	724	94	0.453	0.156	True	DNA mismatch repair protein
PGF_00046029	12.09	293	91	0.707	0.059	True	Pyruvate:ferredoxin oxidoreductase, beta subunit (EC 1.2.7.1)
PGF_00067188	11.80	363	96	0.619	0.092	True	Aspartate-semialdehyde dehydrogenase (EC 1.2.1.11)
PGF_00024323	11.76	364	98	0.617	0.119	True	NAD(P)-dependent glyceraldehyde-3-phosphate dehydrogenase, archaeal (EC 1.2.1.59)
PGF_00006981	11.76	335	95	0.643	0.072	True	GTP cyclohydrolase MptA (EC 3.5.4.39)
PGF_10350547	11.71	396	95	0.589	0.092	True	3-dehydroquinate synthase II (EC 1.4.1.24)
PGF_00121834	11.59	420	93	0.565	0.055	True	hypothetical protein
PGF_08454293	11.58	459	97	0.541	0.113	True	N-acetylglucosamine-1-phosphate uridyltransferase (EC 2.7.7.23) / Glucosamine-1-phosphate N-acetyltransferase (EC 2.3.1.157)
PGF_00067772	11.58	405	98	0.575	0.185	True	[NiFe] hydrogenase metallocenter assembly protein HypE
PGF_02474526	11.56	348	95	0.619	0.061	True	anion-transporting ATPase
PGF_03375688	11.55	340	95	0.626	0.109	True	S-methyl-5-thioribose-1-phosphate isomerase (EC 5.3.1.23)
PGF_00424971	11.48	783	95	0.410	0.269	True	Excinuclease ABC subunit C
PGF_02277678	11.43	423	95	0.556	0.093	True	Phosphoglycerate kinase (EC 2.7.2.3)
PGF_00033954	11.33	366	95	0.592	0.095	True	Phosphoribosylformylglycinamidine cyclo-ligase (EC 6.3.3.1)
PGF_02961521	11.31	656	96	0.442	0.252	True	Fumarate reductase (CoM/CoB), subunit TfrB (EC 1.3.4.1)
PGF_00015737	11.31	372	95	0.586	0.093	True	Isopentenyl-diphosphate delta-isomerase, FMN-dependent (EC 5.3.3.2)
PGF_00413256	11.29	454	91	0.530	0.088	True	tRNA N6-threonylcarbamoyladenosine 2-methylthiotransferase (putative) @ tRNA-i(6)A37 methylthiotransferase (EC 2.8.4.3)
PGF_00853393	11.23	302	95	0.646	0.078	True	Succinyl-CoA ligase [ADP-forming] alpha chain (EC 6.2.1.5)
PGF_00049910	11.18	202	91	0.786	0.028	True	SSU ribosomal protein SAe (S2p)
PGF_00413286	11.16	540	96	0.480	0.238	True	tRNA pseudouridine(13) synthase (EC 5.4.99.27)
PGF_00424925	11.13	274	93	0.672	0.075	True	Eukaryotic translation initiation factor 2 alpha subunit
PGF_00006318	11.05	319	96	0.619	0.094	True	Formylmethanofuran--tetrahydromethanopterin N-formyltransferase (EC 2.3.1.101)
PGF_06674514	11.00	309	94	0.626	0.090	True	Nitrogenase FeMo-cofactor synthesis FeS core scaffold and assembly protein NifB

PGF_00070777	10.89	344	97	0.587	0.043	True	Diphthamide biosynthesis protein 1
PGF_03004613	10.87	495	96	0.488	0.161	True	Histidyl-tRNA synthetase (EC 6.1.1.21)
PGF_00005666	10.83	345	93	0.583	0.057	True	Flap structure-specific endonuclease
PGF_01087653	10.82	314	95	0.611	0.179	True	2-amino-3,7-dideoxy-D-threo-hept-6-ulosonate synthase (EC 2.2.1.10)
PGF_07015581	10.73	426	96	0.520	0.184	True	Carbamoyl-phosphate synthase small chain (EC 6.3.5.5)
PGF_00489714	10.69	369	95	0.557	0.139	True	Porphobilinogen synthase (EC 4.2.1.24)
PGF_00063915	10.69	336	96	0.583	0.090	True	Tyrosyl-tRNA synthetase (EC 6.1.1.1)
PGF_00026805	10.67	334	96	0.584	0.102	True	OB-fold nucleic acid binding domain protein
PGF_06230969	10.63	565	94	0.447	0.130	True	NAD(P)H-hydrate epimerase (EC 5.1.99.6) / ADP-dependent (S)-NAD(P)H-hydrate dehydratase (EC 4.2.1.136)
PGF_00063594	10.63	673	96	0.410	0.252	True	Archaeal DNA polymerase II small subunit (EC 2.7.7.7)
PGF_08376928	10.62	344	95	0.573	0.133	True	Branched-chain amino acid aminotransferase (EC 2.6.1.42)
PGF_02011315	10.62	349	94	0.568	0.077	True	Geranylgeranyl diphosphate synthase (EC 2.5.1.29)
PGF_00421629	10.56	249	96	0.669	0.049	True	DNA polymerase sliding clamp protein PCNA
PGF_00413307	10.56	524	93	0.461	0.209	True	tRNA(Ile)(2)-agmatinylcytidine synthase (EC 6.3.4.22)
PGF_00549380	10.49	300	95	0.606	0.034	True	4-hydroxy-tetrahydrodipicolinate synthase (EC 4.3.3.7)
PGF_00424417	10.46	359	92	0.552	0.094	True	Energy conserving hydrogenase Ehb integral membrane protein O
PGF_00008548	10.45	438	96	0.499	0.219	True	Glycerol-1-phosphate dehydrogenase [NAD(P)+] (EC 1.1.1.261)
PGF_12766951	10.38	386	94	0.529	0.089	True	Phospho-N-acetylmuramoyl-pentapeptide-transferase (EC 2.7.8.13)
PGF_12884281	10.37	428	98	0.501	0.117	True	dNTP triphosphohydrolase, broad substrate specificity
PGF_00413206	10.33	452	94	0.486	0.155	True	tRNA (guanine(26)-N(2))-dimethyltransferase (EC 2.1.1.216)
PGF_00008334	10.31	433	92	0.495	0.115	True	Glutamyl-tRNA reductase (EC 1.2.1.70)
PGF_00000125	10.30	384	97	0.526	0.079	True	7,8-didemethyl-8-hydroxy-5-deazariboflavin synthase subunit 1
PGF_00421665	10.27	581	95	0.426	0.340	True	DNA primase, archaeal DnaG-type
PGF_07889681	10.21	387	95	0.519	0.164	True	N-acetyl-gamma-glutamyl-phosphate reductase (EC 1.2.1.38)
PGF_00424387	10.20	320	93	0.570	0.091	True	Energy conserving hydrogenase Eha associated protein (protein S); Formylmethanofuran--tetrahydromethanopterin N-formyltransferase (EC 2.3.1.101)
PGF_00048585	10.16	262	94	0.627	0.113	True	Exosome complex exonuclease RRP41
PGF_00016613	10.12	328	91	0.559	0.093	True	Lactyl (2) diphospho-(5')guanosine:7,8-didemethyl-8-hydroxy-5-deazariboflavin 2-phospho-L-lactate transferase (EC 2.7.8.28)
PGF_02960239	10.10	459	93	0.471	0.071	True	TldE protein, part of TldE/TldD proteolytic complex
PGF_00033959	10.09	226	93	0.671	0.058	True	Phosphoribosylformylglycinamidine synthase, glutamine amidotransferase subunit (EC 6.3.5.3)
PGF_00064367	10.01	409	95	0.495	0.093	True	UPF0425 pyridoxal phosphate-dependent protein MJ0158
PGF_00051879	9.87	234	95	0.645	0.034	True	Shwachman-Bodian-Diamond syndrome protein; predicted exosome subunit
PGF_02329487	9.87	364	90	0.517	0.074	True	4-[[4-(2-aminoethyl)phenoxy]-methyl]-2-furanmethanamine-glutamate synthase
PGF_10097367	9.81	319	93	0.549	0.105	True	Porphobilinogen deaminase (EC 2.5.1.61)
PGF_00418425	9.66	365	93	0.506	0.215	True	Coenzyme F420 hydrogenase beta subunit (FrcB) (EC 1.12.98.1)
PGF_00415574	9.62	271	93	0.584	0.061	True	FIG00137952: Uncharacterized protein (ATP-grasp superfamily)
PGF_00065257	9.54	273	91	0.577	0.118	True	Uncharacterized protein MJ1676
PGF_00016267	9.48	573	97	0.396	0.339	True	L-tyrosine decarboxylase (EC 4.1.1.25)
PGF_02281324	9.41	424	92	0.457	0.188	True	Uncharacterized protein MJ1086
PGF_00034252	9.39	446	95	0.445	0.142	True	2,5-diamino-6-ribitylamino-pyrimidinone 5-phosphate deaminase, archaeal (EC 3.5.4.-)
PGF_02346669	9.37	358	97	0.495	0.179	True	Ornithine carbamoyltransferase (EC 2.1.3.3)
PGF_00424431	9.28	166	90	0.720	0.114	True	Energy conserving hydrogenase Ehb small subunit (protein M)
PGF_00853991	9.23	312	97	0.522	0.106	True	Diaminopimelate epimerase (EC 5.1.1.7)
PGF_00414927	9.18	560	93	0.388	0.197	True	CCA tRNA nucleotidyltransferase, archaeal type (EC 2.7.7.72)
PGF_00048782	9.16	361	93	0.482	0.202	True	Ribose-phosphate pyrophosphokinase (EC 2.7.6.1)
PGF_00065013	9.16	485	94	0.416	0.393	True	Uncharacterized protein MJ0094
PGF_00424430	9.12	239	91	0.590	0.094	True	Energy conserving hydrogenase Ehb protein Q
PGF_00037061	9.07	275	95	0.547	0.069	True	Exosome complex exonuclease RRP45
PGF_00418427	9.00	335	94	0.492	0.233	True	Coenzyme F420 hydrogenase gamma subunit (FrcG) (EC 1.12.98.1)
PGF_01513745	8.90	243	92	0.571	0.154	True	Proteasome subunit beta (EC 3.4.25.1), archaeal
PGF_00422341	8.90	211	94	0.612	0.118	True	DNA-directed RNA polymerase subunit E' (EC 2.7.7.6)
PGF_00260942	8.89	474	90	0.408	0.252	True	UPF0348 protein MJ0951
PGF_05657253	8.84	245	96	0.565	0.099	True	Ribose-5-phosphate isomerase A (EC 5.3.1.6)

PGF_00016341	8.71	215	95	0.594	0.184	False	LSU ribosomal protein L15e
PGF_00421762	8.71	239	92	0.564	0.032	False	DNA repair and recombination protein RadB
PGF_00013803	8.71	199	95	0.618	0.037	False	Imidazoleglycerol-phosphate dehydratase (EC 4.2.1.19)
PGF_00049839	8.59	133	91	0.745	0.013	False	SSU ribosomal protein S13e (S15p)
PGF_00411462	8.53	235	94	0.557	0.088	False	Box C/D RNA-guided RNA methyltransferase subunit fibrillarin
PGF_00413313	8.49	289	92	0.500	0.160	False	tRNA(Phe) (4-demethylwyosine(37)-C(7)) aminocarboxypropyltransferase (EC 2.5.1.114)
PGF_00625415	8.48	255	94	0.531	0.123	False	7-cyano-7-deazaguanine synthase (EC 6.3.4.20)
PGF_07097873	8.48	351	94	0.453	0.186	False	Ribonuclease Z (EC 3.1.26.11)
PGF_01219411	8.47	350	91	0.453	0.094	False	5-methyltetrahydropteroyltriglutamate--homocysteine methyltransferase (EC 2.1.1.14)
PGF_00413205	8.45	417	97	0.414	0.191	False	tRNA (guanine(10)-N(2))-dimethyltransferase (EC 2.1.1.213)
PGF_00066972	8.41	203	93	0.590	0.112	False	Xanthosine/inosine triphosphate pyrophosphatase
PGF_00413210	8.41	436	95	0.403	0.239	False	tRNA (guanine(37)-N(1))-methyltransferase (EC 2.1.1.228)
PGF_00415581	8.39	224	97	0.561	0.085	False	23S rRNA (uridine(2552)-2'-O)-methyltransferase (EC 2.1.1.166)
PGF_08473931	8.39	199	96	0.595	0.077	False	Flavin prenyltransferase UbiX
PGF_02283727	8.37	145	98	0.695	0.085	False	Eukaryotic translation initiation factor 2 beta subunit
PGF_00049827	8.32	335	98	0.454	0.158	False	SSU rRNA (adenine(1518)-N(6)/adenine(1519)-N(6))-dimethyltransferase (EC 2.1.1.182)
PGF_00418292	8.31	528	97	0.362	0.310	False	Cobalt-precorrin-5B (C1)-methyltransferase (EC 2.1.1.195)
PGF_02925426	8.30	541	92	0.357	0.276	False	Uncharacterized protein MJ0971
PGF_00426932	8.27	149	94	0.677	0.108	False	6,7-dimethyl-8-ribityllumazine synthase (EC 2.5.1.78)
PGF_00048479	8.25	163	92	0.647	0.097	False	Riboflavin synthase archaeal (EC 2.5.1.9)
PGF_08181546	8.24	346	91	0.443	0.201	False	Shikimate 5-dehydrogenase I alpha (EC 1.1.1.25)
PGF_00051543	8.21	381	96	0.421	0.245	False	Shikimate kinase II (EC 2.7.1.71)
PGF_00411463	8.15	462	92	0.379	0.196	False	Box C/D RNA-guided RNA methyltransferase subunit Nop5
PGF_00025580	8.04	190	97	0.583	0.095	False	Nicotinamide-nucleotide adenylyltransferase, NadM family (EC 2.7.7.1)
PGF_00070158	8.04	133	96	0.697	0.025	False	conserved protein associated with acetyl-CoA C-acyltransferase and HMGCo
PGF_01435183	8.02	203	96	0.563	0.078	False	Adenine phosphoribosyltransferase (EC 2.4.2.7)
PGF_03518570	8.01	170	95	0.614	0.136	False	Nucleoside diphosphate kinase (EC 2.7.4.6)
PGF_07544077	8.00	324	95	0.444	0.192	False	Prephenate dehydratase (EC 4.2.1.51)
PGF_04867467	7.99	222	94	0.536	0.103	False	Imidazole glycerol phosphate synthase amidotransferase subunit HisH
PGF_00013510	7.94	236	93	0.517	0.145	False	IMP cyclohydrolase (EC 3.5.4.10) [alternate form]
PGF_00015730	7.91	334	93	0.433	0.222	False	Isopentenyl phosphate kinase (EC 2.7.4.26)
PGF_00049978	7.74	253	93	0.486	0.117	False	2-amino-5-formylamino-6-ribosylaminopyrimidin-4(3H)-one 5-monophosphate deformylase (EC 3.5.1.102)
PGF_00006991	7.57	532	95	0.328	0.529	False	GTP cyclohydrolase III (EC 3.5.4.29)
PGF_00061926	7.42	364	94	0.389	0.225	False	Triphosphoribosyl-dephospho-CoA synthetase
PGF_00416211	7.40	367	93	0.386	0.256	False	Candidate phosphomevalonate decarboxylase; COG1355, Predicted dioxygenase
PGF_00065006	7.37	744	97	0.270	0.490	False	Uncharacterized protein MJ0065
PGF_00066960	7.32	181	91	0.544	0.139	False	Aspartate carbamoyltransferase regulatory chain (PyrI)
PGF_00016426	7.30	91	92	0.765	0.029	False	LSU ribosomal protein L37Ae
PGF_00418309	7.27	280	95	0.434	0.279	False	Cobalt-precorrin-8 methylmutase (EC 5.4.99.60)
PGF_00060434	7.21	127	93	0.640	0.188	False	Translation initiation factor 1A
PGF_02781328	7.21	338	91	0.392	0.232	False	NAD synthetase (EC 6.3.1.5)
PGF_02278091	7.16	423	94	0.348	0.401	False	Serine/threonine-protein kinase RIO1 (EC 2.7.11.1)
PGF_00016438	7.11	112	91	0.672	0.183	False	LSU ribosomal protein L44e
PGF_00070870	7.08	244	95	0.453	0.199	False	eRF1 methyltransferase catalytic subunit MTQ2
PGF_02278032	7.05	219	91	0.476	0.120	False	Uncharacterized C4-type Zn finger protein MJ0530
PGF_00424939	7.04	169	92	0.542	0.252	False	Eukaryotic translation initiation factor 5A
PGF_02191019	7.02	486	95	0.318	0.320	False	Thiamine-monophosphate kinase (EC 2.7.4.16)
PGF_00413290	6.96	409	91	0.344	0.325	False	tRNA pseudouridine(38-40) synthase (EC 5.4.99.12)
PGF_00055077	6.92	230	90	0.456	0.206	False	Sulfopyruvate decarboxylase - beta subunit (EC 4.1.1.79)
PGF_00413204	6.85	249	98	0.434	0.288	False	tRNA (cytidine(56)-2'-O)-methyltransferase (EC 2.1.1.206)
PGF_00049898	6.80	149	94	0.557	0.158	False	SSU ribosomal protein S6e
PGF_00016363	6.77	99	96	0.681	0.031	False	LSU ribosomal protein L21e
PGF_00056896	6.66	271	91	0.405	0.196	False	Thymidylate synthase (EC 2.1.1.45)
PGF_00049905	6.65	141	90	0.560	0.134	False	SSU ribosomal protein S8e

PGF_04359948	6.61	864	96	0.225	0.620	False	Selenophosphate synthetase-related protein MJ0640
PGF_00424416	6.54	229	92	0.432	0.279	False	Energy conserving hydrogenase Ehb ferredoxin-containing protein L
PGF_01513918	6.51	224	93	0.435	0.193	False	Adenylate cyclase (EC 4.6.1.1)
PGF_04815798	6.39	630	96	0.255	0.492	False	Mevalonate kinase (EC 2.7.1.36)
PGF_00049859	6.35	242	90	0.408	0.407	False	SSU ribosomal protein S19e
PGF_02776739	6.29	161	95	0.496	0.181	False	Phosphoribosyl-AMP cyclohydrolase (EC 3.5.4.19)
PGF_00065054	6.14	141	92	0.517	0.139	False	Uncharacterized protein MJ0408
PGF_00046039	6.09	201	92	0.430	0.268	False	Pyruvoyl-dependent arginine decarboxylase (EC 4.1.1.19)
PGF_00065076	6.07	219	94	0.410	0.301	False	Uncharacterized protein MJ0498
PGF_00083887	5.84	129	91	0.515	0.307	False	hypothetical protein
PGF_00016417	5.83	115	90	0.544	0.234	False	LSU ribosomal protein L34e
PGF_00067769	5.80	92	95	0.604	0.099	False	[NiFe] hydrogenase metallocenter assembly protein HypC
PGF_02976460	5.78	380	91	0.297	0.494	False	Phosphatidylinositol phosphate synthase @ Archaetidylinositol phosphate synthase (EC 2.7.8.39)
PGF_00067788	5.73	670	92	0.222	0.674	False	[NiFe] hydrogenase nickel incorporation-associated protein HypB
PGF_07957805	5.60	93	90	0.581	0.086	False	Phosphoribosylformylglycinamidine synthase, PurS subunit (EC 6.3.5.3)
PGF_02278971	3.47	296	93	0.202	0.668	False	PaaD-like protein MJ1129

Phylogenetic Tree Report

Rendered Tree

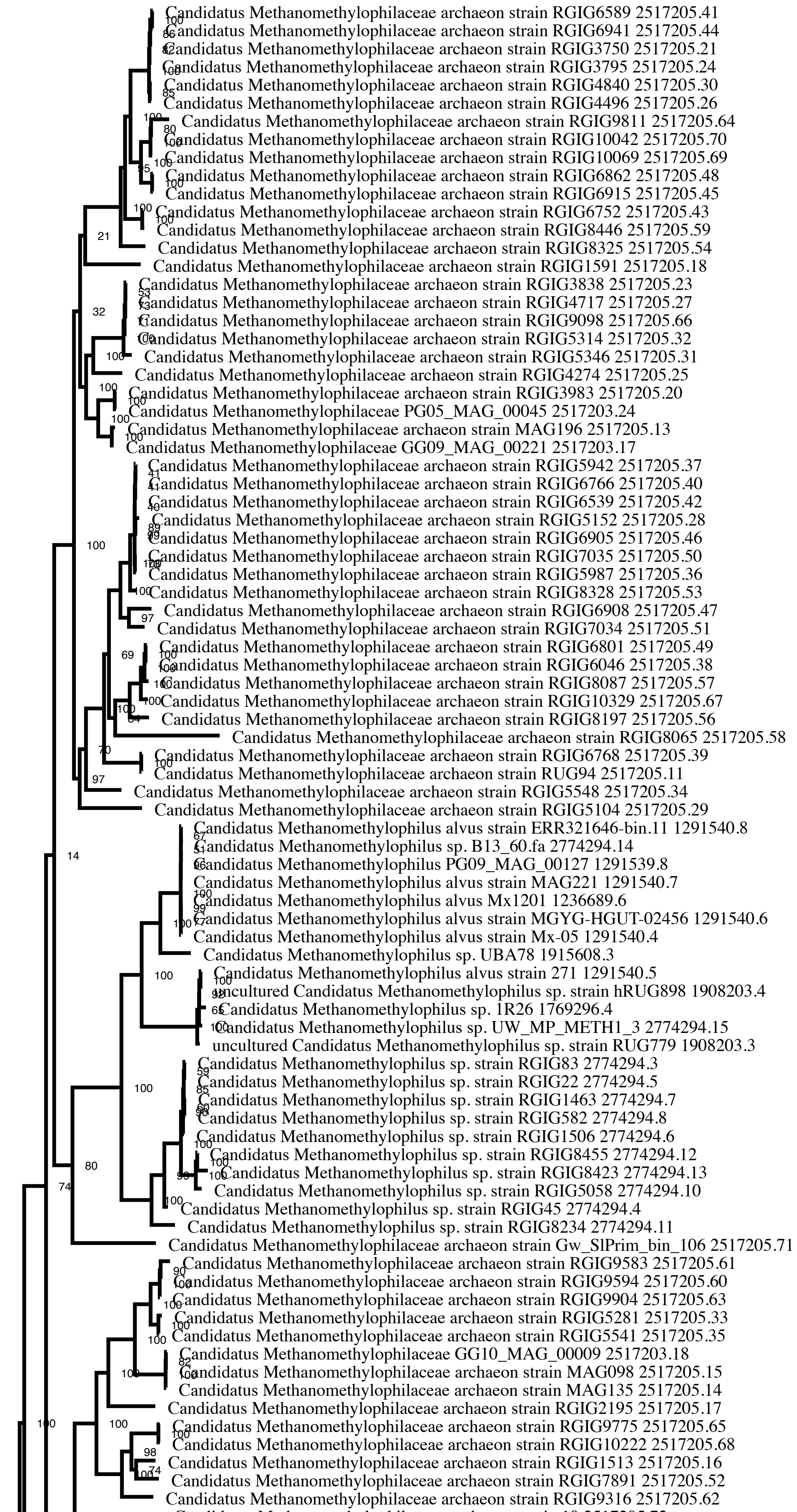

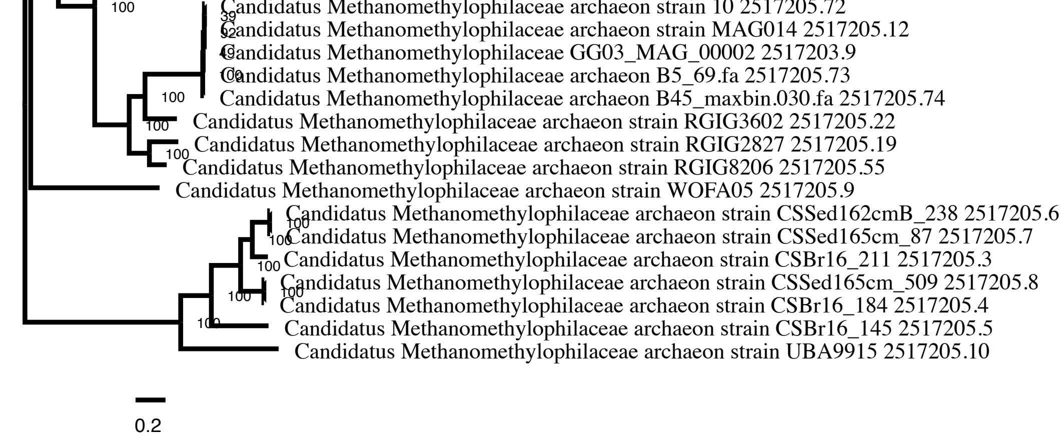

Alternate View

Files with more details

Tree Analysis Statistics

**Requested genomes**	100
**Genomes with data**	99
**Genomes lacking data**	1
**Max allowed deletions**	10
**Max allowed duplications**	10
**Single-copy genes requested**	100
**Single-copy genes found**	7
**Num protein alignments**	7
**Alignment program**	mafft
**Protein alignment time**	145.2 seconds
**Num aligned amino acids**	3041
**Num CDS alignments**	7
**Num aligned nucleotides**	9123
**Best protein model found by RAxML**	LG
**Branch support method**	RAxML Fast Bootstrapping
**RAxML likelihood**	-258394.3039
**RAxML version**	8.2.11
**RAxML time**	9361.9 seconds
**Total time**	9601.1 seconds

RAxML Command Line

Goal: Analyze proteins with model 'AUTO' to find best substitution model.
raxmlHPC-PTHREADS-SSE3 -s Methanomethylophilaceae_10_MAX_DELs_DUPs_proteins.phy -n Methanomethylophilaceae_10_MAX_DELs_DUPs_proteins -m PROTCATAUTO -p 12345 -T 12 -e 10
Process time: 1060.386 seconds

Goal: Find best tree.
raxmlHPC-PTHREADS-SSE3 -s Methanomethylophilaceae_10_MAX_DELs_DUPs.phy -n Methanomethylophilaceae_10_MAX_DELs_DUPs -m GTRCAT -q Methanomethylophilaceae_10_MAX_DELs_DUPs.partitions -p 12345 -T 12 -f a -x 12345 -N 100
Process time: 8301.561 seconds

RAxML Codon and Amino Acid Partitions

```
DNA, codon1 = 1-9123\3
DNA, codon2 = 2-9123\3
DNA, codon3 = 3-9123\3
LG, proteins = 9124-12164
```

Genome Statistics

GenomeId	Total Genes	Single Copy	Used	Name
2517205.71	177	4	4	Candidatus Methanomethylophilaceae archaeon strain Gw_SlPrim_bin_106
2517205.60	193	5	5	Candidatus Methanomethylophilaceae archaeon strain RGIG9594
2517205.19	260	7	7	Candidatus Methanomethylophilaceae archaeon strain RGIG2827
2517205.59	261	5	5	Candidatus Methanomethylophilaceae archaeon strain RGIG8446
2517205.52	263	7	7	Candidatus Methanomethylophilaceae archaeon strain RGIG7891
2517205.67	266	4	4	Candidatus Methanomethylophilaceae archaeon strain RGIG10329
2774294.8	270	6	6	Candidatus Methanomethylophilus sp. strain RGIG582
2774294.10	270	7	7	Candidatus Methanomethylophilus sp. strain RGIG5058
2517205.57	274	5	5	Candidatus Methanomethylophilaceae archaeon strain RGIG8087
2517205.8	275	4	4	Candidatus Methanomethylophilaceae archaeon strain CSSed165cm_509
2517205.33	277	5	5	Candidatus Methanomethylophilaceae archaeon strain RGIG5281
2517205.65	288	5	5	Candidatus Methanomethylophilaceae archaeon strain RGIG9775
2517205.68	291	5	5	Candidatus Methanomethylophilaceae archaeon strain RGIG10222
2517205.64	293	6	6	Candidatus Methanomethylophilaceae archaeon strain RGIG9811
2517205.63	294	7	7	Candidatus Methanomethylophilaceae archaeon strain RGIG9904
2517205.55	307	6	6	Candidatus Methanomethylophilaceae archaeon strain RGIG8206
2517205.30	311	5	5	Candidatus Methanomethylophilaceae archaeon strain RGIG4840
2517205.31	314	6	6	Candidatus Methanomethylophilaceae archaeon strain RGIG5346
2517205.28	317	6	6	Candidatus Methanomethylophilaceae archaeon strain RGIG5152
2517205.62	323	7	7	Candidatus Methanomethylophilaceae archaeon strain RGIG9316
2517205.61	326	6	6	Candidatus Methanomethylophilaceae archaeon strain RGIG9583
2774294.3	329	6	6	Candidatus Methanomethylophilus sp. strain RGIG83
2517205.7	330	4	4	Candidatus Methanomethylophilaceae archaeon strain CSSed165cm_87
2517205.3	331	7	7	Candidatus Methanomethylophilaceae archaeon strain CSBr16_211
2517205.56	334	6	6	Candidatus Methanomethylophilaceae archaeon strain RGIG8197
2517205.21	334	5	5	Candidatus Methanomethylophilaceae archaeon strain RGIG3750
2517205.20	335	5	5	Candidatus Methanomethylophilaceae archaeon strain RGIG3983
2517205.50	355	7	7	Candidatus Methanomethylophilaceae archaeon strain RGIG7035
2517205.5	356	3	3	Candidatus Methanomethylophilaceae archaeon strain CSBr16_145
2517205.10	361	7	7	Candidatus Methanomethylophilaceae archaeon strain UBA9915
2517205.9	364	5	5	Candidatus Methanomethylophilaceae archaeon strain WOFA05
2517205.69	366	6	6	Candidatus Methanomethylophilaceae archaeon strain RGIG10069
2517205.23	367	6	6	Candidatus Methanomethylophilaceae archaeon strain RGIG3838
2517203.17	374	6	6	Candidatus Methanomethylophilaceae GG09_MAG_00221
2517205.40	377	5	5	Candidatus Methanomethylophilaceae archaeon strain RGIG6766
2517205.53	380	6	6	Candidatus Methanomethylophilaceae archaeon strain RGIG8328
2517205.70	383	6	6	Candidatus Methanomethylophilaceae archaeon strain RGIG10042
2517205.49	397	7	7	Candidatus Methanomethylophilaceae archaeon strain RGIG6801
2517205.4	397	7	7	Candidatus Methanomethylophilaceae archaeon strain CSBr16_184
2517205.73	400	7	7	Candidatus Methanomethylophilaceae archaeon B5_69.fa
2517205.17	407	7	7	Candidatus Methanomethylophilaceae archaeon strain RGIG2195
2517205.29	408	7	7	Candidatus Methanomethylophilaceae archaeon strain RGIG5104
2517205.24	409	5	5	Candidatus Methanomethylophilaceae archaeon strain RGIG3795
2774294.4	412	7	7	Candidatus Methanomethylophilus sp. strain RGIG45
2517205.11	415	7	7	Candidatus Methanomethylophilaceae archaeon strain RUG94
2517205.54	418	7	7	Candidatus Methanomethylophilaceae archaeon strain RGIG8325
2517205.46	420	7	7	Candidatus Methanomethylophilaceae archaeon strain RGIG6905
2517205.41	425	6	6	Candidatus Methanomethylophilaceae archaeon strain RGIG6589
2517205.44	427	7	7	Candidatus Methanomethylophilaceae archaeon strain RGIG6941
2517205.22	430	6	6	Candidatus Methanomethylophilaceae archaeon strain RGIG3602
2517205.72	431	7	7	Candidatus Methanomethylophilaceae archaeon strain 10
2517205.18	431	7	7	Candidatus Methanomethylophilaceae archaeon strain RGIG1591
2517203.9	432	7	7	Candidatus Methanomethylophilaceae GG03_MAG_00002
2517205.58	434	4	4	Candidatus Methanomethylophilaceae archaeon strain RGIG8065

2517205.15	438	6	6	Candidatus Methanomethylophilaceae archaeon strain MAG098
2517205.74	440	7	7	Candidatus Methanomethylophilaceae archaeon B45_maxbin.030.fa
2517205.35	441	7	7	Candidatus Methanomethylophilaceae archaeon strain RGIG5541
2517205.14	441	7	7	Candidatus Methanomethylophilaceae archaeon strain MAG135
2517205.34	444	7	7	Candidatus Methanomethylophilaceae archaeon strain RGIG5548
2517205.12	444	7	7	Candidatus Methanomethylophilaceae archaeon strain MAG014
2774294.5	445	7	7	Candidatus Methanomethylophilus sp. strain RGIG22
2517205.38	445	7	7	Candidatus Methanomethylophilaceae archaeon strain RGIG6046
2774294.7	454	7	7	Candidatus Methanomethylophilus sp. strain RGIG1463
2517205.48	455	7	7	Candidatus Methanomethylophilaceae archaeon strain RGIG6862
2517205.51	455	7	7	Candidatus Methanomethylophilaceae archaeon strain RGIG7034
2517205.6	456	7	7	Candidatus Methanomethylophilaceae archaeon strain CSSed162cmB_238
2517205.43	458	7	7	Candidatus Methanomethylophilaceae archaeon strain RGIG6752
2517205.42	467	7	7	Candidatus Methanomethylophilaceae archaeon strain RGIG6539
2517205.26	467	7	7	Candidatus Methanomethylophilaceae archaeon strain RGIG4496
2517205.32	473	7	7	Candidatus Methanomethylophilaceae archaeon strain RGIG5314
2517205.37	473	7	7	Candidatus Methanomethylophilaceae archaeon strain RGIG5942
2517205.45	475	7	7	Candidatus Methanomethylophilaceae archaeon strain RGIG6915
2517205.25	475	7	7	Candidatus Methanomethylophilaceae archaeon strain RGIG4274
2774294.11	480	7	7	Candidatus Methanomethylophilus sp. strain RGIG8234
2517203.18	484	7	7	Candidatus Methanomethylophilaceae GG10_MAG_00009
2517205.13	486	7	7	Candidatus Methanomethylophilaceae archaeon strain MAG196
2517205.47	488	7	7	Candidatus Methanomethylophilaceae archaeon strain RGIG6908
2774294.13	488	7	7	Candidatus Methanomethylophilus sp. strain RGIG8423
2517205.66	488	7	7	Candidatus Methanomethylophilaceae archaeon strain RGIG9098
2517205.39	492	7	7	Candidatus Methanomethylophilaceae archaeon strain RGIG6768
2774294.6	496	7	7	Candidatus Methanomethylophilus sp. strain RGIG1506
2517205.16	500	7	7	Candidatus Methanomethylophilaceae archaeon strain RGIG1513
2517205.36	501	7	7	Candidatus Methanomethylophilaceae archaeon strain RGIG5987
2517205.27	502	7	7	Candidatus Methanomethylophilaceae archaeon strain RGIG4717
2774294.12	536	7	7	Candidatus Methanomethylophilus sp. strain RGIG8455
2517203.24	543	7	7	Candidatus Methanomethylophilaceae PG05_MAG_00045
1291539.8	1128	7	7	Candidatus Methanomethylophilus PG09_MAG_00127
2774294.15	1155	7	7	Candidatus Methanomethylophilus sp. UW_MP_METH1_3
1291540.7	1163	7	7	Candidatus Methanomethylophilus alvus strain MAG221
2774294.14	1187	6	6	Candidatus Methanomethylophilus sp. B13_60.fa
1915608.3	1208	7	7	Candidatus Methanomethylophilus sp. UBA78
1908203.3	1237	7	7	uncultured Candidatus Methanomethylophilus sp. strain RUG779
1769296.4	1294	7	7	Candidatus Methanomethylophilus sp. 1R26
1908203.4	1308	7	7	uncultured Candidatus Methanomethylophilus sp. strain hRUG898
1291540.5	1308	7	7	Candidatus Methanomethylophilus alvus strain 271
1291540.8	1350	7	7	Candidatus Methanomethylophilus alvus strain ERR321646-bin.11
1291540.4	1448	7	7	Candidatus Methanomethylophilus alvus strain Mx-05
1291540.6	1462	7	7	Candidatus Methanomethylophilus alvus strain MGYG-HGUT-02456
1236689.6	1504	7	7	Candidatus Methanomethylophilus alvus Mx1201
2774294.9	0	0	0	Deleted

Gene Family Statistics

Gene families are ranked by alignment score combining mean per-position variability, alignment length, and gappiness.

PGFam	Align. Score	Align. Length	Num Seqs	Mean Sqr Freq	Prop Gaps	Used In Analysis	Product
PGF_00421879	15.08	699	88	0.571	0.127	True	DNA topoisomerase VI subunit B (EC 5.99.1.3)
PGF_00020796	13.58	578	93	0.565	0.146	True	Methyl coenzyme M reductase system component A2

PGF_00421761	13.52	319	89	0.757	0.078	True	DNA repair and recombination protein RadA
PGF_00013509	12.83	606	89	0.521	0.242	True	IMP cyclohydrolase (EC 3.5.4.10) / Phosphoribosylaminoimidazolecarboxamide formyltransferase (EC 2.1.2.3)
PGF_00007012	11.66	502	90	0.521	0.269	True	GTP-binding and nucleic acid-binding protein YchF
PGF_00020791	10.25	210	93	0.708	0.088	True	Methyl coenzyme M reductase operon protein C
PGF_00049827	9.85	311	89	0.559	0.185	True	SSU rRNA (adenine(1518)-N(6)/adenine(1519)-N(6))-dimethyltransferase (EC 2.1.1.182)

Strategies to Increase Single-Copy Gene Number

Number of single-copy genes (7) was less than requested (100).
Examining the number of genes per genome in the 'Genome Statistics' table above may indicate incomplete or plasmid entries with few genes which may be removed.
Criteria for calling single copy genes can be made more lenient by increasing Max Allowed Deletions and/or Max Allowed Duplications.

Omitting one of the following sets of genomes will provide the approximate boost in single-copy genes:

scGene Boost	Genomes to Omit
1	2517205.21 2517205.22 2517205.28 2517205.30 2517205.40 2517205.49 2517205.5 2517205.53 2517205.56 2517205.59 2517205.61 2517205.64 2517205.7
1	2517203.17 2517205.28 2517205.31 2517205.32 2517205.49 2517205.57 2517205.58 2517205.60 2517205.65 2517205.67 2517205.71 2774294.10 2774294.3 2774294.4 2774294.5 2774294.7 2774294.8
1	2517205.13 2517205.19 2517205.3 2517205.30 2517205.41 2517205.5 2517205.59 2517205.62 2517205.63 2517205.7 2517205.9 2774294.10
1	2517205.41 2517205.53 2517205.60 2517205.64 2517205.65 2517205.68 2517205.69 2517205.7 2517205.71
1	2517205.11 2517205.13 2517205.19 2517205.22 2517205.28 2517205.3 2517205.35 2517205.37 2517205.4 2517205.51 2517205.52 2517205.54 2517205.55 2517205.57 2517205.59 2517205.60 2517205.62 2517205.8 2774294.10 2774294.3 2774294.7
