# Supplementary figures and images for: Novel complete methanogenic pathways in longitudinal genomic study of monogastric age-associated archaea
Source: Anim Microbiome. 2023 Jul 17;5:35. doi: 10.1186/s42523-023-00256-6 (PMC10353118; doi:10.1186/s42523-023-00256-6)

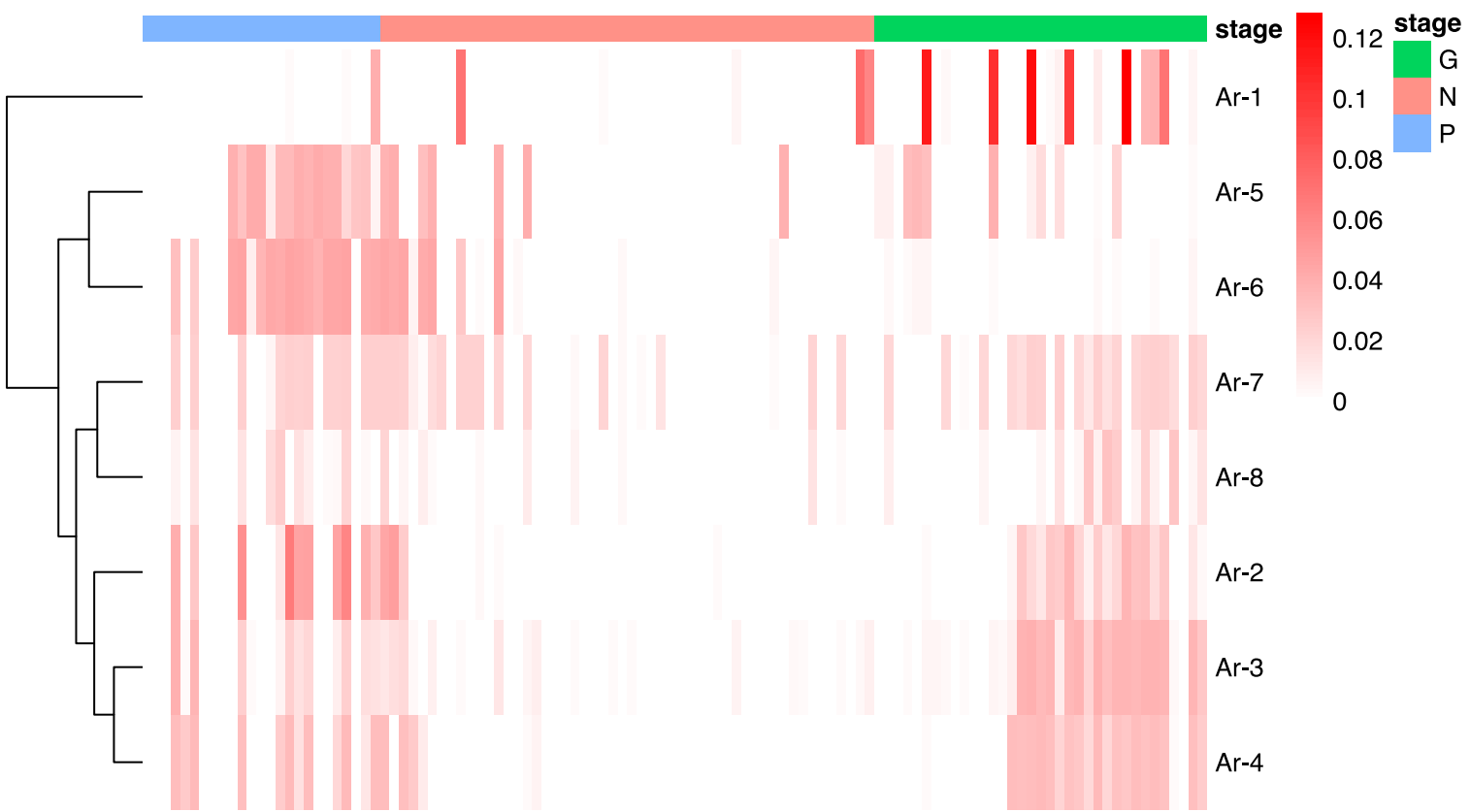

Supplement: Supplementary file 4 — Additional file 4: Fig. S2: Relative abundance of archaea-MAGs (rows) across all individual sample metagenomes (columns) (Preweaning [P]; nursery [N]; growth adult [G]). [file 42523_2023_256_MOESM4_ESM.pdf]
